# A Vegetable Diet

**Published:** 1875-06

**Authors:** 


### A VEGETABLE DIET.

Whether we are disposed to accept the doctrine that a strictly vegetable diet is conducive to long life and the perfect physical condition which attends longevity, or not, we can not fail to be interested in all the speculations and arguments upon the subject. These arguments are numerous, and many of them are very hard to answer. It is known that the diseases from which the wealthiest classes suffer, are gouty and rheumatic diseases,—diseases which are known as being of a "strumous" and "lithic-acid" character; and it is well nigh impossible to attribute them to anything except an imperfect assimilation of food; in other words, to an excess of meat-diet. The most important adjunct in the cure of this class of very painful ailments is found in a diet of fruits and vegetables, to the total exclusion of flesh. It is found, also, that those who eat least of meat, recover the most speedily from wounds and severe accidents. The shock of losing a limb by being run over by a railroad-train, will be recovered from ten times by vegetable-eaters where only one meat-eater will recover. The vegetable-eating China-man will recover from almost any injury which is not instantly mortal.

It is claimed, too, that vegetarians have more exemption from the attacks of epidemic disease than flesh-eaters; in particular, it is denied that any case of cholera has been found among them. It is also admitted by physiologists in general that the cases of extreme longevity are almost solely found among vegetarians. Of course, many things must conspire that an individual may attain the greatest age possible to man. He must have had no hereditary weakness, no violent shock from accident or acute disease, no permanent excess of toil or distressing care, no long exposure to bad atmosphere in cities; and, if vegetarian food is of critical importance, he must have been a vegetarian from childhood; then possibly he will live to the age a hundred. It is ridiculous to expect that by adopting this practice late in life an individual can become signal in longevity; yet it is maintained he may somewhat lengthen his years, especially because the diet itself suffices to cure many maladies, probably by the greater purity which it gives to the blood. The case of Prof. Adam Ferguson is signal and notorious. When past fifty he was seized with very alarming paralysis. His friend, Dr. Black, the celebrated discoverer of latent heat, who was no vegetarian, was called in to treat him, and prescribed a strict vegetarian and milk diet. Under this he entirely recovered; ate no meat and drank only water or weak tea for the rest of his life; had no second attack; and, after the age of seventy was remarkably hearty, continuing in much vigor until almost ninety. He lived to ninety-three. The effect of a mere vegetarian diet to renew shattered life appears here undeniable. Fruit, which is presumed to have been the food of original man—of man who is born "a tropical product," with hairless body—fruit is to him peculiarly medicine as well as food. The Germans have their "grape-cure," and, among fruits, let grapes by all means have a most honorable mention; yet happily they do not stand alone.

When a child was covered with ulcers from head to foot, and blinded by them — when physicians despaired and confessed drugs to be useless— Mr. S. Rowbotham, a Surgeon of Stockport, guiltless of vegetarian theory, cured the patient perfectly in a few months, by a diet of stewed English fruit and honey.

To pass from these details of experience to the higher region of comparative anatomy and physiology, it is contended that the interior organs and teeth of man show him to have been made for a frugivorous animal. To this day physicians of eminence may be heard to say that our canine teeth show us to be made for tearing flesh. That this is a gross error is no new discovery. Linnæus, Gassendi, Ray, Cuvier, Thomas Bell, Lawrence, equally with Prof. Richard Owen, avow our teeth not to be canine, but to be nearest to apes' teeth. Their fangs are indeed larger than ours, and well adapted to crack strong nutshells; but none of them in a state of nature eat flesh. Indeed, any one who examines a dog's teeth, sees at once the entire contrast; yet our scientific men (so called) allow the epithet *canine* to run away with them! A close comparison of the digestive organs in man with those of the domestic animals on the one side, and of the carnivora on the other, shows distinctly that his organs are *intermediate*, as are those of the apes. The entire argument is very extensive. Mr. John Smith develops it in his "Fruits and Farinacea." Here it can only be pointed at. He admits that art and the use of fire make flesh tolerable to us as food, but denies that that which art enables us to do can ever thereby become normally necessary, or tend to so great robustness as the use of that food to which our physiology and anatomy direct us. The mediate place between herbivorous and carnivorous animals is denoted by the epithet *frugiverous.* This is the place of man, also of the apes and monkeys; apparently, too, of the bear and the pig. The horse also has some approximation to the human organs, perhaps because grain is a food so well suited to him. In detail, certain peculiarities are alleged, to which a reader (if he concedes the facts to be all correct) will give what weight he thinks they deserve. It is said that no carnivorous animals sweat, but all herbivorous animals sweat; and, since man sweats, this allies him more closely to the herbivora. It is further said, that in the carnivora the salivary glands are comparatively small, in the herbivora very large; and the reason too is plain. The herbivora masticate their food with their broad grinders, and need saliva for the operation; but the carnivora never grind food; they have no grinders, and they can not masticate. Now, in all these points man resembles the herbivora.

---

Every worthy young man should be made to feel, that he may honorably offer his heart and hand to any woman, whenever he is able *with her assistance*, to provide a home with the necessaries of life and ordinary comforts; and every young woman should be taught to regard it as neither derogatory to her character nor social position, to accept such an offer, and to help provide such a home.

